# Supplementary material for: Dynamic switching from coherent perfect absorption to parametric amplification in a nonlinear spoof plasmonic waveguide
Source: Nat Commun. 2024 Apr 1;15:2824. doi: 10.1038/s41467-024-47191-x (PMC11271628; doi:10.1038/s41467-024-47191-x)
Supplement: Supplementary file 1 — Supplementary information [file 41467_2024_47191_MOESM1_ESM.pdf]

Supplementary Information for

Dynamic switching from coherent perfect absorption to parametric amplification in a nonlinear spoof plasmonic waveguide

Wen Yi Cui[1,2], Jingjing Zhang[1,2,*], Yu Luo[3,*], Xinxin Gao[4], and Tie Jun Cui[1,2,*]

1 State Key Laboratory of Millimeter Waves, Southeast University, Nanjing 210096, China

2 Institute of Electromagnetic Space, Southeast University, Nanjing 210096, China.

3 Key Laboratory of Radar Imaging and Microwave Photonics, Nanjing University of Aeronautics and Astronautics, Nanjing 211106, China

4 State Key Laboratory of Terahertz and Millimeter Waves, City University of Hong Kong, Hong Kong SAR, China

* Correspondence should be addressed to Jingjing Zhang, Yu Luo and Tie Jun Cui.

Emails: zhangjingjing@seu.edu.cn; yu.luo@nuaa.edu.cn; tjcui@seu.edu.cn

This supplementary information contains the following sections:

Supplementary Note 1. Theoretical analysis of nonlinear CPA

Supplementary Note 2. Phase matching frequency and linewidth of the device

Supplementary Note 3. Determination of the optimal waveguide length

Supplementary Note 4. Influence of the pump and signal power on signal gain

Supplementary Note 5. Using a low-frequency pump to control a high-frequency signal

Supplementary Note 1. Theoretical analysis of nonlinear CPA

Assuming that the waves propagate along the z direction, then the electric fields of signal and pump waves can be respectively expressed as:

$$E_s(z) = A_s(z)\mathrm{e}^{\mathrm{i}k_s z - \mathrm{i}\omega_s t},\ E_p(z) = A_p(z)\mathrm{e}^{\mathrm{i}k_p z - \mathrm{i}\omega_p t},$$

where A_s, A_p, ω_s, ω_p, k_s and k_p are respectively the electric field amplitudes, frequencies and wave numbers of the signal and pump waves. The wave number k is divided into real part k' representing the propagation phase and imaginary part k'' representing the transmission loss. That is to say, $k_s = k_s' + \mathrm{i}k_s''$ and $k_p = k_p' + \mathrm{i}k_p''$. The electric field amplitudes and frequencies satisfy the following relations:

$$\frac{\mathrm{d}A_s(z)}{\mathrm{d}z} = \mathrm{i}\frac{\omega_s^2}{k_s c_0^2}\chi_{\mathrm{eff}}^{(2)} A_p(z) A_s^*(z)\mathrm{e}^{-\mathrm{i}\Delta k' z}\mathrm{e}^{-k_p'' z}, \tag{S1a}$$

$$\frac{\mathrm{d}A_p(z)}{\mathrm{d}z} = \mathrm{i}\frac{\omega_p^2}{2k_p c_0^2}\chi_{\mathrm{eff}}^{(2)} A_s^2(z)\mathrm{e}^{\mathrm{i}\Delta k' z}\mathrm{e}^{-(2k_s'' - k_p'')z}, \tag{S1b}$$

in which $\Delta k' = 2k_s' - k_p'$ indicates the phase mismatch, c_0 is the light velocity, and $\chi_{\mathrm{eff}}^{(2)}$ is the second-order effective nonlinear coefficient. We consider the solution in the nondepletion pump regime, where $A_p(z)$ is a constant (complex) value and $\frac{\mathrm{d}A_p(z)}{\mathrm{d}z} \approx 0$ so $A_p(z)$ can be written as A_p. In this case, the partial derivative with respect to z and the complex conjugation of Eq. (S1a) are written as

$$\frac{\mathrm{d}^2 A_s(z)}{\mathrm{d}z^2} = \mathrm{i}\frac{\omega_s^2}{k_s c_0^2}\chi_{\mathrm{eff}}^{(2)} A_p \mathrm{e}^{\mathrm{i}(\mathrm{i}k_p'' - \Delta k')z}\frac{\mathrm{d}A_s^*(z)}{\mathrm{d}z} - (\mathrm{i}k_p'' - \Delta k')\frac{\omega_s^2}{k_s c_0^2}\chi_{\mathrm{eff}}^{(2)} A_p A_s^*(z)\mathrm{e}^{\mathrm{i}(\mathrm{i}k_p'' - \Delta k')z}, \tag{S2a}$$

$$\frac{\mathrm{d}A_s^*(z)}{\mathrm{d}z} = -\mathrm{i}\frac{\omega_s^2}{k_s^* c_0^2}\chi_{\mathrm{eff}}^{(2)*} A_p^* A_s(z)\mathrm{e}^{\mathrm{i}\Delta k' z}\mathrm{e}^{-k_p'' z}. \tag{S2b}$$

Substituting Eq. (S1a) and Eq. (S2b) into Eq. (S2a) yields,

$$\frac{\mathrm{d}^2 A_s(z)}{\mathrm{d}z^2} + (k_p'' + \mathrm{i}\Delta k')\frac{\mathrm{d}A_s(z)}{\mathrm{d}z} - \frac{\omega_s^4 \left|\chi_{\mathrm{eff}}^{(2)}\right|^2 \left|A_p\right|^2}{\left|k_s\right|^2 c_0^4}\mathrm{e}^{-2k_p'' z} A_s(z) = 0. \tag{S3}$$

In the lossless case $k_p'' = 0$, the general solution of Eq. (S3) takes the form of

$$A_s(z)=A_s(0)\mathrm{e}^{\frac{-\mathrm{i}\Delta k'}{2}}\left(C_1\mathrm{e}^{\sqrt{\gamma^2-\frac{\Delta k'^2}{4}}}+C_2\mathrm{e}^{-\sqrt{\gamma^2-\frac{\Delta k'^2}{4}}}\right), \tag{S4a}$$

where $\gamma=\frac{\omega_s^2}{c_0^2}\left|\frac{\chi_{\mathrm{eff}}^{(2)}A_p}{k_s}\right|$. In the lossy case $k_p''\neq 0$, the general solution of Eq. (S3) is obtained as

$$A_s(z)=A_s(0)\mathrm{e}^{-\alpha k_p'' z}\left[C_1\,\mathrm{I}_\alpha\left(\beta\mathrm{e}^{-k_p'' z}\right)+C_2\,\mathrm{K}_\alpha\left(\beta\mathrm{e}^{-k_p'' z}\right)\right], \tag{S4b}$$

where we have introduced two constants $\alpha=\frac{1}{2}+\mathrm{i}\frac{\Delta k'}{2k_p''}$ and $\beta=\frac{\omega_s^2}{k_p'' c_0^2}\left|\frac{\chi_{\mathrm{eff}}^{(2)}A_p}{k_s}\right|$, $\mathrm{I}_\alpha(\bullet)$ and $\mathrm{K}_\alpha(\bullet)$ are respectively the α-th order modified Bessel functions of the first and the second kinds, and C_1 and C_2 can be determined by the initial conditions at z = 0. Performing the partial derivative of Eq. (S4b) to z, we get:

$$\frac{\mathrm{d}A_s(z)}{\mathrm{d}z}=-A_s(0)k_p''\mathrm{e}^{-\alpha k_p'' z}\{C_1[\alpha\,\mathrm{I}_\alpha(\beta\mathrm{e}^{-k_p'' z})+\beta\,\mathrm{I}'_\alpha(\beta\mathrm{e}^{-k_p'' z})]+C_2[\alpha\mathrm{K}_\alpha(\beta\mathrm{e}^{-k_p'' z})+\beta\,\mathrm{K}'_\alpha(\beta\mathrm{e}^{-k_p'' z})]\}.$$

Substituting the above equation into Eq. (S1a) yields

$$\begin{aligned}&\mathrm{i}\frac{\omega^2}{k_s c_0^2}\chi_{\mathrm{eff}}^{(2)}A_p A_s^*(z)\mathrm{e}^{-\mathrm{i}\Delta k' z}\mathrm{e}^{-k_p'' z}\\ &=-A_s(0)k_p''\mathrm{e}^{-\alpha k_p'' z}\{C_1[\alpha\,\mathrm{I}_\alpha(\beta\mathrm{e}^{-k_p'' z})+\beta\,\mathrm{I}'_\alpha(\beta\mathrm{e}^{-k_p'' z})]+C_2[\alpha\mathrm{K}_\alpha(\beta\mathrm{e}^{-k_p'' z})+\beta\mathrm{K}'_\alpha(\beta\mathrm{e}^{-k_p'' z})]\}.\end{aligned} \tag{S5}$$

When $z=0$, Eq. (S4b) and Eq. (S5) can be respectively reduced to

$$1=C_1\,\mathrm{I}_\alpha(\beta)+C_2\,\mathrm{K}_\alpha(\beta), \tag{S6a}$$

$$-\mathrm{i}\frac{\omega_s^2\chi_{\mathrm{eff}}^{(2)}A_p}{k_p'' k_s c_0^2}\frac{A_s^*(0)}{A_s(0)}=C_1\left[\alpha\,\mathrm{I}_\alpha(\beta)+\beta\,\mathrm{I}'_\alpha(\beta)\right]+C_2\left[\alpha\,\mathrm{K}_\alpha(\beta)+\beta\,\mathrm{K}'_\alpha(\beta)\right]. \tag{S6b}$$

Setting the initial phases of the signal wave and pump wave as φ_s and φ_p, then $A_s(0)$ and constant A_p can be expressed as $|A_s|\mathrm{e}^{\mathrm{i}\varphi_s}$ and $|A_p|\mathrm{e}^{\mathrm{i}\varphi_p}$, and $A_s^*(0)=|A_s|\mathrm{e}^{-\mathrm{i}\varphi_s}$. Thus we can solve C_1 and C_2 to be

$$C_1 = \frac{\mathrm{i}\frac{\omega_s^2 \chi_{\mathrm{eff}}^{(2)} |A_p|}{k_p'' k_s c_0^2} \mathrm{K}_\alpha(\beta) \mathrm{e}^{\mathrm{i}(\varphi_p - 2\varphi_s)} + \alpha \mathrm{K}_\alpha(\beta) + \beta \mathrm{K}'_\alpha(\beta)}{\beta[\mathrm{I}_\alpha(\beta)\mathrm{K}'_\alpha(\beta) - \mathrm{I}'_\alpha(\beta)\mathrm{K}_\alpha(\beta)]}$$

$$C_2 = -\frac{\mathrm{i}\frac{\omega_s^2 \chi_{\mathrm{eff}}^{(2)} |A_p|}{k_p'' k_s c_0^2} \mathrm{I}_\alpha(\beta) \mathrm{e}^{\mathrm{i}(\varphi_p - 2\varphi_s)} + \alpha \mathrm{I}_\alpha(\beta) + \beta \mathrm{I}'_\alpha(\beta)}{\beta[\mathrm{I}_\alpha(\beta)\mathrm{K}'_\alpha(\beta) - \mathrm{I}'_\alpha(\beta)\mathrm{K}_\alpha(\beta)]}. \quad \text{(S7)}$$

Now we have got

$$E_s(z) = A_s(z)\mathrm{e}^{\mathrm{i}k_s z - \mathrm{i}\omega_s t} = A_s(0)\mathrm{e}^{-\alpha k_p'' z}\left[C_1 \mathrm{I}_\alpha\left(\beta \mathrm{e}^{-k_p'' z}\right) + C_2 \mathrm{K}_\alpha\left(\beta \mathrm{e}^{-k_p'' z}\right)\right]\mathrm{e}^{\mathrm{i}k_s' z - k_s'' z - \mathrm{i}\omega_s t}.$$

The signal gain after propagating across a distance *L* can be obtained

$$G = \left|\frac{E_s(L)}{E_s(0)}\right|^2$$

$$= \left| \frac{\left(\alpha + \mathrm{i}\frac{\omega_s^2 \chi_{\mathrm{eff}}^{(2)} |A_p|}{k_p'' k_s c_0^2} \mathrm{e}^{\mathrm{i}(\varphi_p - 2\varphi_s)}\right)\left[\mathrm{K}_\alpha(\beta)\mathrm{I}_\alpha\left(\beta \mathrm{e}^{-k_p'' L}\right) - \mathrm{I}_\alpha(\beta)\mathrm{K}_\alpha\left(\beta \mathrm{e}^{-k_p'' L}\right)\right] + \beta[\mathrm{K}'_\alpha(\beta)\mathrm{I}_\alpha\left(\beta \mathrm{e}^{-k_p'' L}\right) - \mathrm{I}'_\alpha(\beta)\mathrm{K}_\alpha\left(\beta \mathrm{e}^{-k_p'' L}\right)]}{\beta[\mathrm{I}_\alpha(\beta)\mathrm{K}'_\alpha(\beta) - \mathrm{I}'_\alpha(\beta)\mathrm{K}_\alpha(\beta)]} \right|^2 \mathrm{e}^{-2\left(\alpha k_p'' + k_s''\right)L}. \quad \text{(S8)}$$

At the phase-matching points where $\Delta k' = 0 \Rightarrow \alpha = \frac{1}{2}$, Eq. (S8) can be reduced to

$$G = \left| \frac{\left(\frac{1}{2} + \mathrm{i}\frac{\omega_s^2 \chi_{\mathrm{eff}}^{(2)} |A_p|}{k_p'' k_s c_0^2} \mathrm{e}^{\mathrm{i}\varphi_p - 2\mathrm{i}\varphi_s}\right)\left[\mathrm{K}_{\frac{1}{2}}(\beta)\mathrm{I}_{\frac{1}{2}}\left(\beta \mathrm{e}^{-k_p'' L}\right) - \mathrm{I}_{\frac{1}{2}}(\beta)\mathrm{K}_{\frac{1}{2}}\left(\beta \mathrm{e}^{-k_p'' L}\right)\right] + \beta[\mathrm{K}'_{\frac{1}{2}}(\beta)\mathrm{I}_{\frac{1}{2}}\left(\beta \mathrm{e}^{-k_p'' L}\right) - \mathrm{I}'_{\frac{1}{2}}(\beta)\mathrm{K}_{\frac{1}{2}}\left(\beta \mathrm{e}^{-k_p'' L}\right)]}{\beta[\mathrm{I}_{\frac{1}{2}}(\beta)\mathrm{K}'_{\frac{1}{2}}(\beta) - \mathrm{I}'_{\frac{1}{2}}(\beta)\mathrm{K}_{\frac{1}{2}}(\beta)]} \right|^2 \mathrm{e}^{-\left(k_p'' + 2k_s''\right)L}. \quad \text{(S9)}$$

Here, the propagation distance L is just the length of SSPP waveguide. In experiments, we cannot measure $\chi_{\mathrm{eff}}^{(2)}$ and $|A_p|$ respectively, but we can calculate $\chi_{\mathrm{eff}}^{(2)}|A_p|$ by measuring the signal gain.

Supplementary Note 2. Phase matching frequency and linewidth of the device

If we fix the pump frequency at the phase matching frequency point and vary the input signal frequency, we will only observe a single peak/dip of the signal gain. However, if we fix the relationship of $f_p = 2f_s$, instead of fixing f_p, there is indeed a linewidth (blue shaded region), as shown in Supplementary Fig. 1. The measured 3dB bandwidth is about 0.036GHz and the corresponding quality factor $Q = 2\pi\frac{4.46}{0.036} \approx 778$).

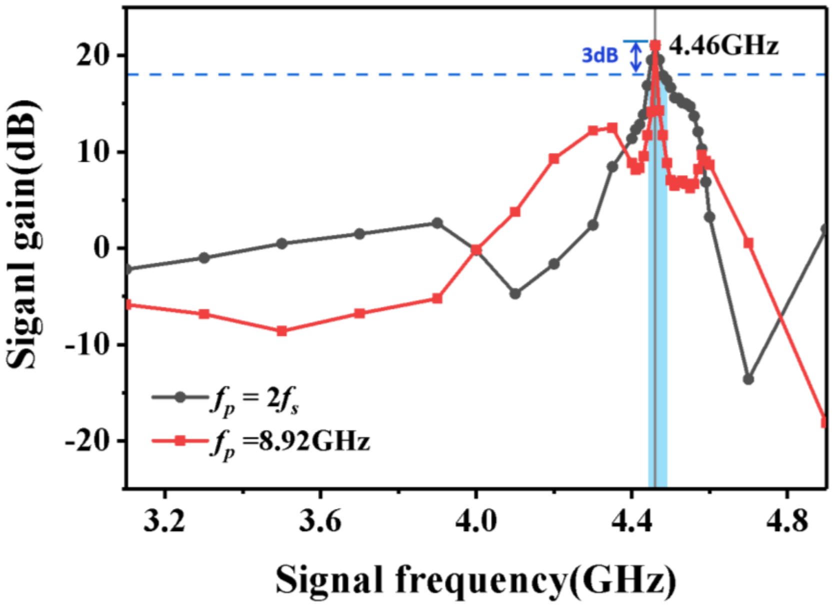

Supplementary Fig. 1 | The measured signal gains versus the signal frequencies at $f_p = 2f_s$ and f_p = 8.92GHz. The unit number of the SSPP waveguide is N = 60.

The factors influencing the linewidth and the associated limiting factor Q are the phase mismatch $\Delta k' = 2k_s' - k_p'$ affected by the shape of the dispersion curve and the propagation length L. Here, we set $\Delta k' = \gamma\Delta\omega_s$, and vary the constant γ from $0.5\gamma_0$ to $1.5\gamma_0$ to qualitatively calculate the linewidth and Q versus the waveguide length under different $\Delta k'$. Supplementary Fig. 2 verifies that, as the phase mismatch or waveguide length increases, the

operation linewidth decreases and the Q factor increases. Therefore, to achieve a large operation linewidth, the dispersion curve must be well designed to keep the phase mismatch (out of the phase matching frequency) small and the optimal waveguide length short.

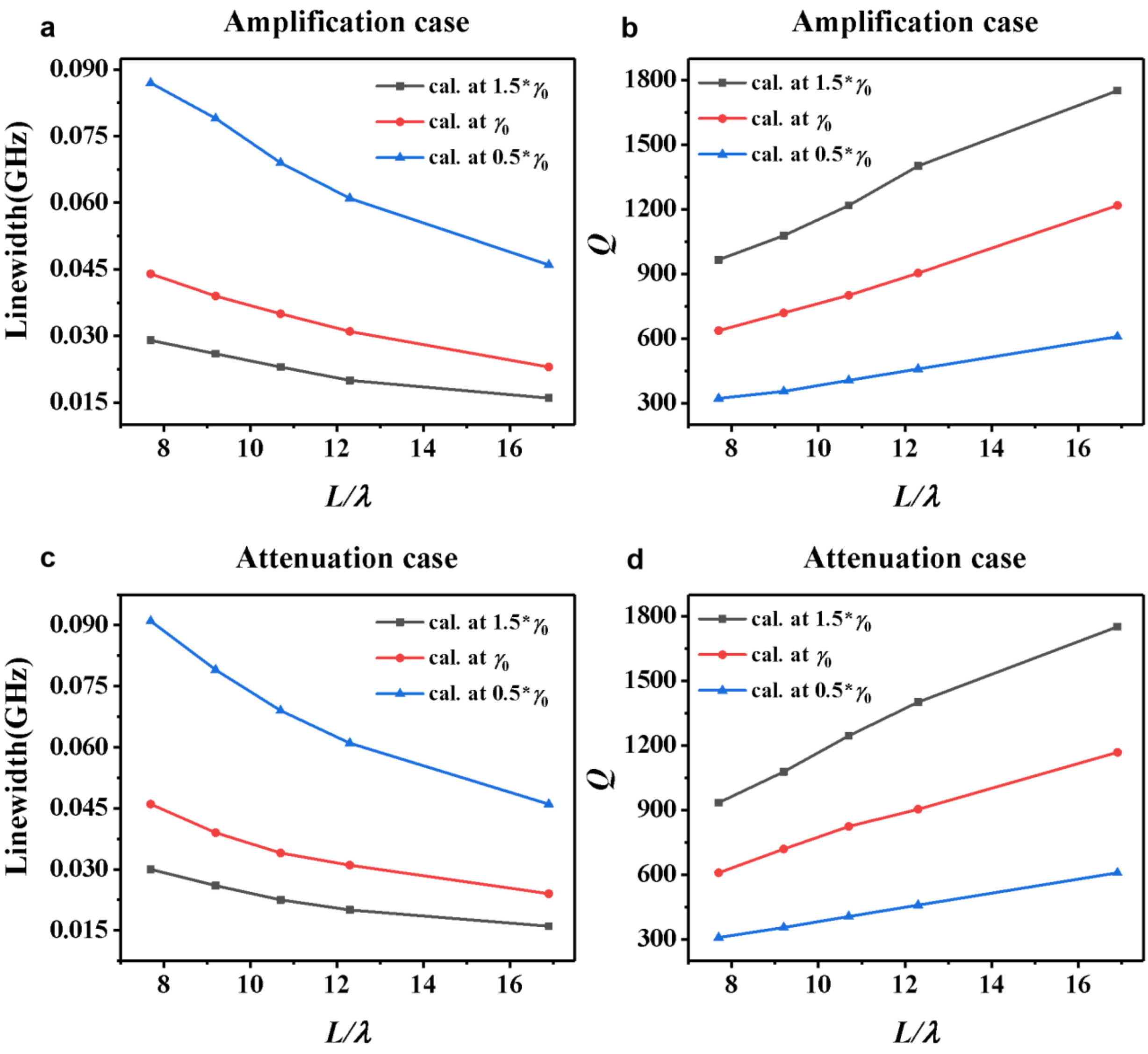

Supplementary Fig. 2 | The calculated operation linewidth and Q factor versus the waveguide length L/λ under different γ when $f_p = 2f_s$. **a** The linewidth in the amplification case. **b** The Q factor in the amplification case. **c** The linewidth in the attenuation case. **d** The Q factor in the attenuation case.

Supplementary Note 3. Determination of the optimal waveguide length

In an ideal lossless case, the gain of the signal wave will increase monotonically with the propagation length under the phase matching condition in the PA cases. However, in the

presence of the loss, there is a critical length at which the gain reaches the peak value. Note that the gain remains relatively stable (within 2dB variation) around the peak in a certain range of propagation length, as highlighted in the figures. As the propagation length further increases, the loss will dominate and the total gain will gradually decrease. So the loss tangent determined by the substrate material and nonlinear elements will affect k_s'' and k_p'', and thus the robustness of waveguide length. In addition, the $\chi_{\text{eff}}^{(2)}$ determined by the nonlinear elements also has a direct effect on the robustness. Here, we plot signal gain as the function of the propagation length *L*, and investigate how k_s'', k_p'', and $\chi_{\text{eff}}^{(2)}$ would affect the robustness, as shown in Supplementary Fig. 3. In the PA case, we can observe that the maximum signal gain as well as the robustness weaken with larger k_s'', or k_p'' but strengthen with $\chi_{\text{eff}}^{(2)}$.

Meanwhile, the attenuation increases monotonically with the propagation length in the CPA cases, as shown in the Supplementary Fig. 3. Here, we mark the critical length at which the attenuation reaches 30 dB (corresponding to 99.9% attenuation). Note that the robustness for attenuation strengthens with the increase of k_s'' or $\chi_{\text{eff}}^{(2)}$, but weakens with k_p''.

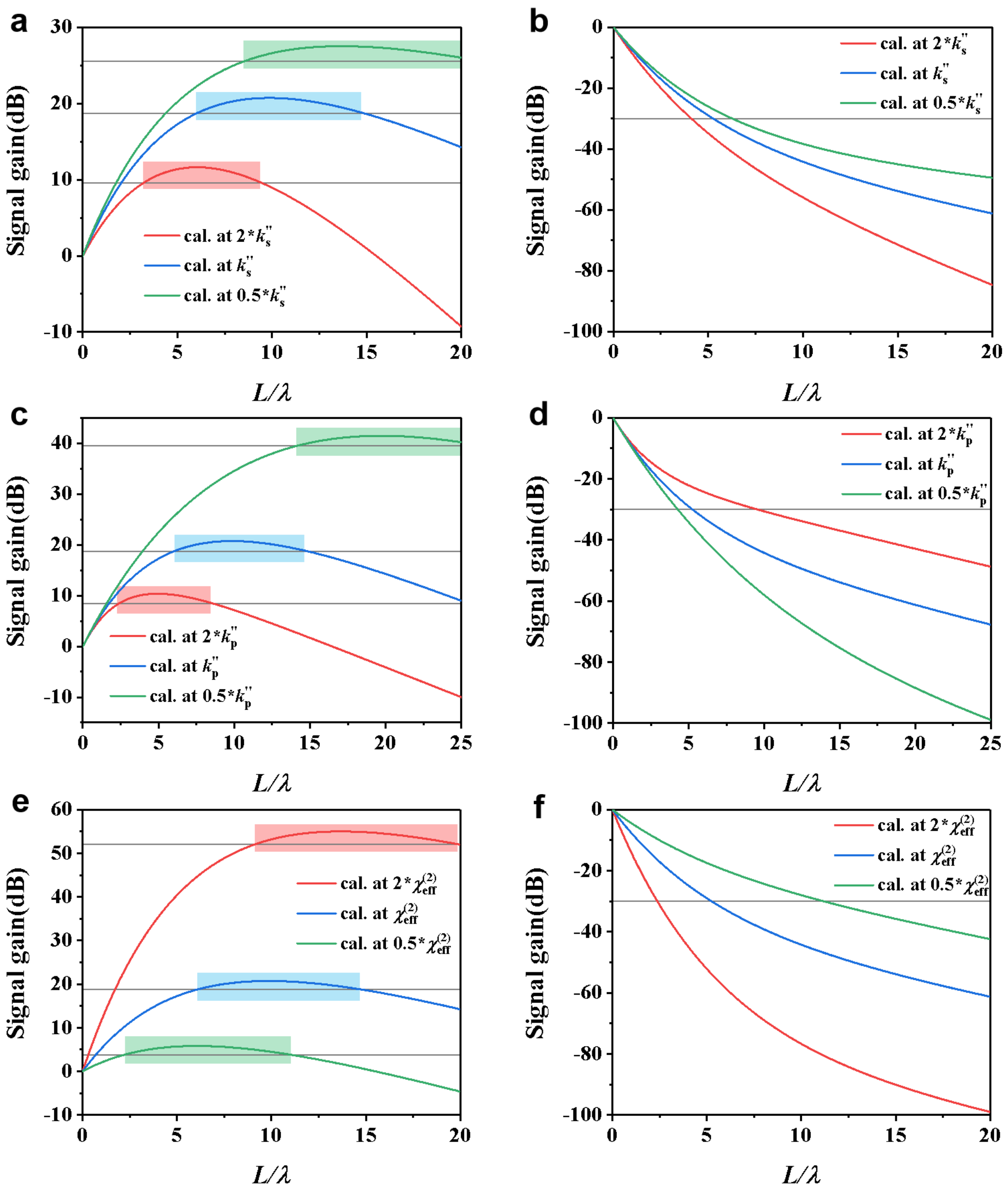

Supplementary Fig. 3 | The signal gains versus the propagation length (L/λ) in the cases of PA ($\varphi_p - 2\varphi_s = -90^\circ + \arg(k_s)$) and CPA ($\varphi_p - 2\varphi_s = 90^\circ + \arg(k_s)$). **a** PA with different k_s''. **b** CPA with different k_s''. **c** PA with different k_p''. **d** CPA with different k_p''. **e** PA with different $\chi_{\text{eff}}^{(2)}$. **f** CPA with different $\chi_{\text{eff}}^{(2)}$.

Supplementary Note 4. Influence of the pump and signal power on signal gain

From Eq. (3), we note that under the undepletion pump approximation, the pump power affects the signal gain but the signal power does not. However, as the signal power increases, the undepletion approximation is no longer valid, and the signal power starts to have influence on the signal gain, causing the measured signal gain to deviate from the calculated results.

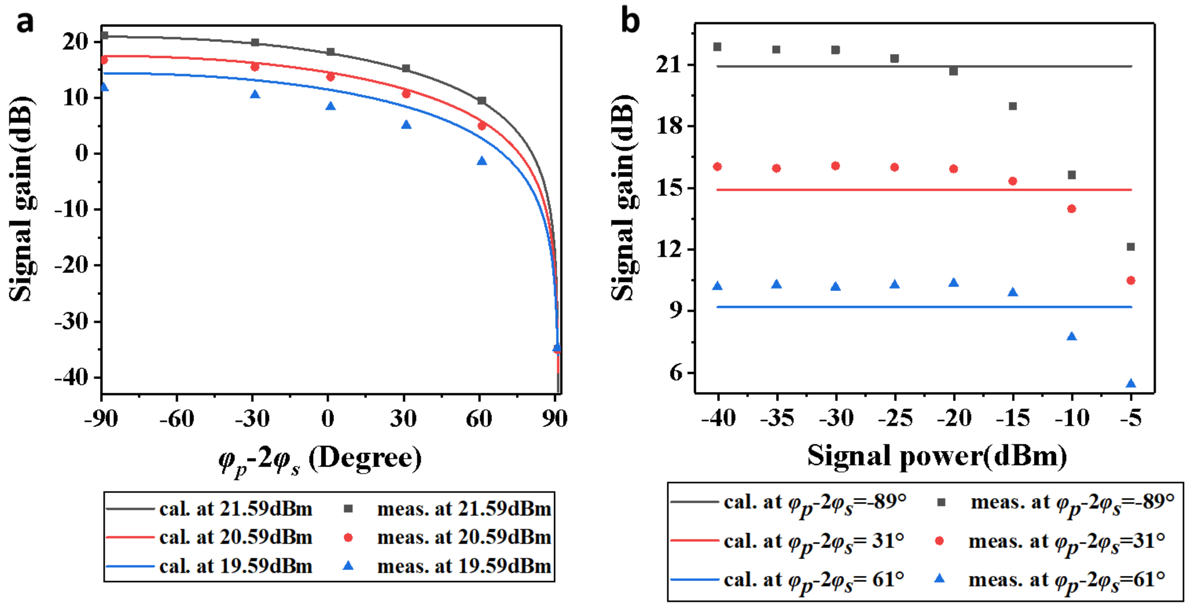

Supplementary Fig. 4 | The signal gains when the transmission distance is $L = 9.2\lambda$. **a** The signal gains versus $\varphi_p - 2\varphi_s$ at different pump powers. **b** The signal gains versus the signal power at different $\varphi_p - 2\varphi_s$ under 21.59dBm pump power.

Due to experimental limitations, the maximum pump power we can use is about 21.6dBm (the use of power amplifier will generate a component at the signal frequency) and I only measured the signal gains at lower pump power. We can observe from Supplementary Fig. 4a that the signal gain is sensitive to the pump power. The lower the pump power is, the smaller the maximum gain and absorption will be. Theoretically, a 2dB decrease in pump power can reduce both the maximum gain and attenuation by about 6dB. We also calculated the signal gain at extremely high and low pump power in Supplementary Fig. 5 to show the effect of power on modulation range more clearly. When $|A_p|$ is large enough, we can use a very short

nonlinear waveguide to obtain high gain and perfect absorption. Supplementary Fig. 4b illustrates that the signal gain will finally fall as the signal power reach a specific value. In order to achieve largest modulation depth, the signal power needs to be below -25dBm when the pump power is 21.59dBm. In other words, for the undepleted pumped approximation to work, the signal wave should remain 4 order of magnitude smaller than the pumped one.

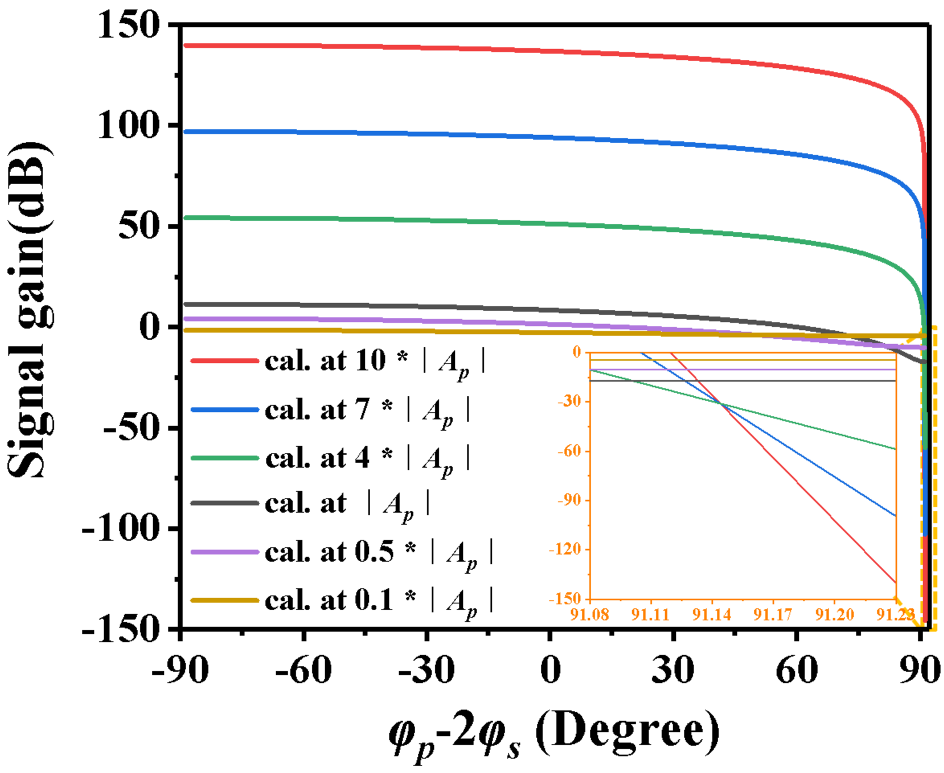

Supplementary Fig. 5 | The signal gains versus the phase difference $\varphi_p - 2\varphi_s$ when transmission distance $L = 2.5\lambda$.

Supplementary Note 5. Using a low-frequency pump to control a high-frequency signal

If we exchange the frequency of the signal wave and the pump wave, we can still use the high-power low-frequency pump wave to control the low-power high-frequency signal wave. In this case, $\omega_s = 2\omega_p$ and $k_s' = 2k_p'$. Consider the co-propagating signal and pump waves with the electric fields given by $E_s(z) = A_s(z)\mathrm{e}^{\mathrm{i}(k_s'+\mathrm{i}k_s'')z-2\mathrm{i}\omega_p t}$ and $E_p(z) = A_p(z)\mathrm{e}^{\mathrm{i}(k_p'+\mathrm{i}k_p'')z-\mathrm{i}\omega_p t}$. When

$A_p(z) >> A_s(z)$, we can assume that $A_p(z)$ is undepleted and $\frac{\mathrm{d}A_p(z)}{\mathrm{d}z} \approx 0$, so $A_p(z)$ can be written as A_p. In this case, the nonlinear coupled mode equation can be written as

$$\frac{\mathrm{d}A_s(z)}{\mathrm{d}z} = \frac{2\mathrm{i}\omega_p^2}{k_s c_0^2} \chi_{\mathrm{eff}}^{(2)} A_p^2 \mathrm{e}^{-\mathrm{i}\Delta kz}, \tag{S10}$$

where $\Delta k = (k_s' - 2k_p') + \mathrm{i}(k_s'' - 2k_p'') = \mathrm{i}(k_s'' - 2k_p'')$. The general solution of the equation above can be found as,

$$A_s(z) = \mathrm{i}\frac{2\omega_p^2}{(k_s'' - 2k_p'')k_s c_0^2} \chi_{\mathrm{eff}}^{(2)} A_p^2 \mathrm{e}^{-\mathrm{i}\Delta kz} + C, \tag{S11}$$

where the constant C can be determined by the initial conditions at $z = 0$:

$$A_s(0) = \mathrm{i}\frac{2\omega_p^2 \chi_{\mathrm{eff}}^{(2)} A_p^2}{(k_s'' - 2k_p'')k_s c_0^2} + C \;\Rightarrow\; C = A_s(0) - \mathrm{i}\frac{2\omega_p^2 \chi_{\mathrm{eff}}^{(2)} A_p^2}{(k_s'' - 2k_p'')k_s c_0^2}. \tag{S12}$$

Now we have got

$$E_s(z) = A_s(z)\mathrm{e}^{\mathrm{i}k_s' - k_s'' z - 2\mathrm{i}\omega_p t}. \tag{S13}$$

The signal gain can be calculated as,

$$G = \left|\frac{E_s(L)}{E_s(0)}\right|^2 = \left|1 + \mathrm{i}\frac{2\omega_p^2 \chi_{\mathrm{eff}}^{(2)} A_p^2}{(2k_p'' - k_s'')k_s c_0^2 A_s(0)}\left(1 - e^{-\mathrm{i}\Delta kL}\right)\right|^2 \mathrm{e}^{-2k_s'' L}. \tag{S14}$$

Setting the initial phases of the signal wave and pump wave as φ_s and φ_p, then $A_s(0)$ and constant A_p can be expressed as $|A_s|\mathrm{e}^{\mathrm{i}\varphi_s}$ and $|A_p|\mathrm{e}^{\mathrm{i}\varphi_p}$, so the signal gain can be written as

$$G = \left|1 + \mathrm{i}\frac{2\omega_p^2 \chi_{\mathrm{eff}}^{(2)} |A_p|^2}{(2k_p'' - k_s'')k_s c_0^2 |A_s|} \mathrm{e}^{\mathrm{i}(2\varphi_p - \varphi_s)} \left(1 - \mathrm{e}^{-\mathrm{i}\Delta kL}\right)\right|^2 \mathrm{e}^{-2k_s'' L}. \tag{S15}$$

Comparison of the Eq. (S15) with Eq. (3) in the manuscript shows that the biggest difference from the case of $\omega_p = 2\omega_s$ is that, when $\omega_s = 2\omega_p$, in addition to $|A_p|$, $|A_s|$ also

has an effect on the gain. Thus, by making the input signal power small enough, a weak pump wave is also able to control the signal wave, which is not possible in $\omega_p = 2\omega_s$ case.

We draw the gain/absorption of the signal wave in terms of the propagation distance L/λ and loss tangent of the nonlinear medium or signal power in $\omega_s = 2\omega_p$ case in Supplementary Fig. 6. It shows that for a fixed length nonlinear waveguide, its loss tangent must be a specific value to achieve CPA. In contrast, the $\omega_p = 2\omega_s$ case is insensitive to the loss tangent and can achieve CPA at many lengths. Supplementary Fig. 6 also illustrates that the input signal power affects the signal gain, which implies that the signal power that can be fully absorbed on different lengths of waveguide varies at the same pump power.

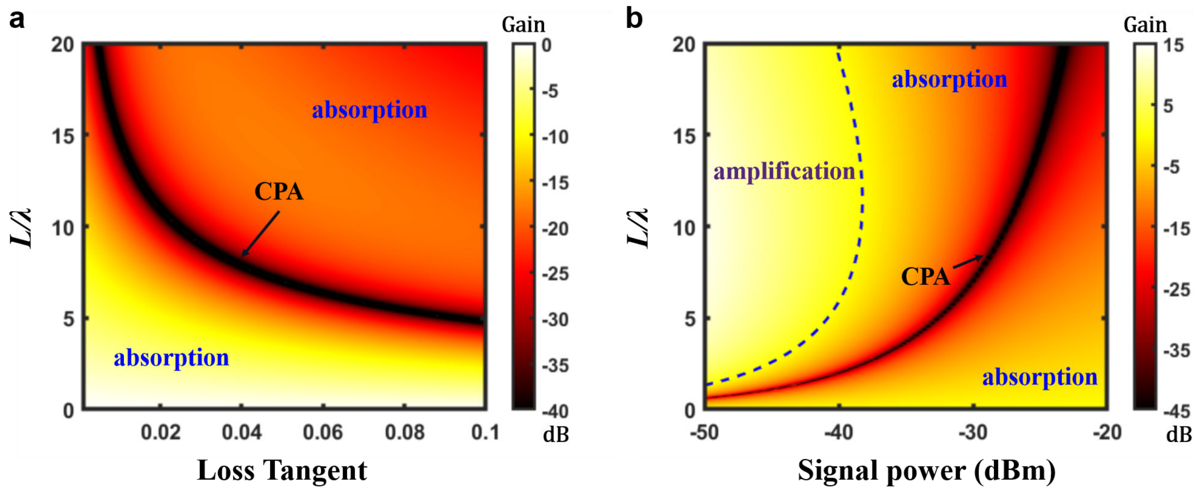

Supplementary Fig. 6 | The amplification and absorption of the signal wave in terms of the propagation L/λ when $\omega_s = 2\omega_p$. **a** The loss tangent of the nonlinear medium. **b** The signal power.

The Eq. (S15) demonstrates that the phase difference between the input waves $2\varphi_p - \varphi_s$ will influence the signal gain. Similar to the $\omega_p = 2\omega_s$ case, the maximum attenuation is achieved at $2\varphi_p - \varphi_s = 90^\circ + \arg(k_s)$ when $\omega_s = 2\omega_p$, in which $\arg(k_s) \approx 0.829^\circ \approx 1^\circ$. As shown in the Supplementary Fig. 7, we measured the signal gain versus the input signal power as well as the phase difference $2\varphi_p - \varphi_s$ when the pump power is about -1.73 dBm and the waveguide length is 9.2λ, and the measured results are in good agreement with the calculated results. The dip in Supplementary Fig. 7a is very sharp so the CPA can only be achieved at

specific signal powers. In contrast, in $\omega_p = 2\omega_s$ case signal waves at any input power can be perfectly absorbed as long as in the nondepletion pump regime. In addition, from the Supplementary Fig. 7 we can see that tuning the phase difference can adjust the signal attenuation, but the modulation range is not large enough for realizing perfect transmission. In conclusion, controlling a high-frequency wave with a low-frequency wave is not as widely applicable as controlling a low-frequency wave with a high-frequency wave.

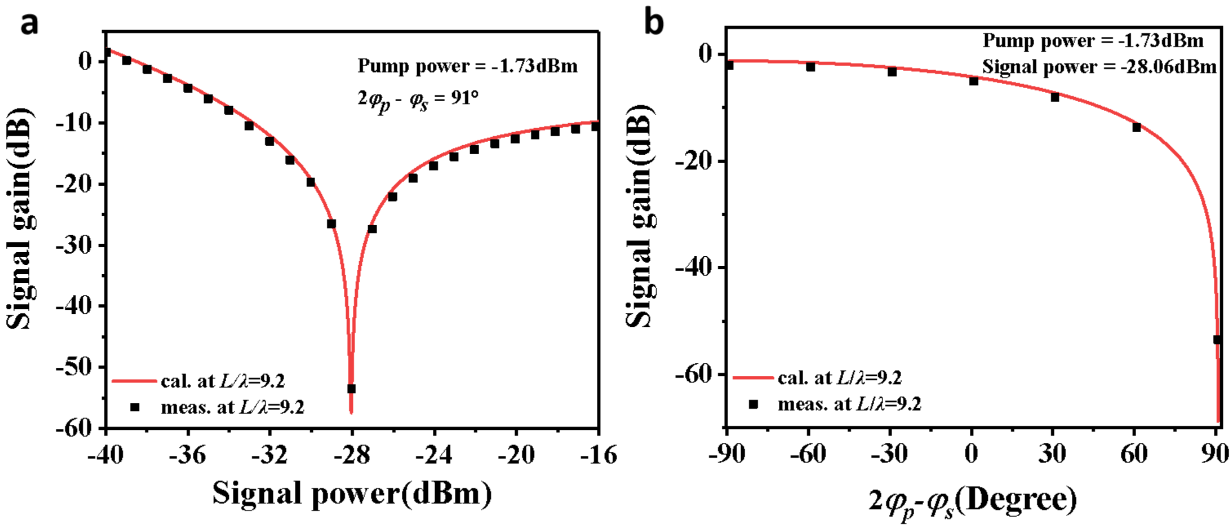

Supplementary Fig. 7 | The calculated and measured signal gains of the nonlinear SSPP waveguide. **a** The signal gains versus the signal power. **b** The signal gains versus the phase difference between the signal and pump waves.
